# La matério-vigilance dans un Centre Hospitalo-Universitaire du Centre-Est tunisien: enquête auprès des médecins

**DOI:** 10.11604/pamj.2016.25.260.10291

**Published:** 2016-12-30

**Authors:** Mohamed Mahjoub, Maher Jedidi, Tasnim Masmoudi, Nabiha Bouafia, Mansour Njah

**Affiliations:** 1 Service d’Hygiène Hospitaliére, CHU Farhat Hached, Sousse, Tunisie; 2 Service de Médecine Légale, CHU Farhat Hached, Sousse, Tunisie

**Keywords:** Médecins, dispositifs médicaux, matério-vigilance, connaissances, attitudes, pratiques, Physicians, medical devices, device-vigilance, knowledge, attitudes, procedures

## Abstract

**Introduction:**

Pour la meilleure gestion des risques en milieu hospitalier et l’amélioration de la qualité et la sécurité de nos soins, le CHU de Sousse (Tunisie), a mis en place, suite aux recommandations de l’ANCSEP (Agence Nationale de Contrôle Sanitaire et Environnementale des Produits) un système de matério-vigilance (MV). En Tunisie l’absence d’un cadre réglementaire organisant la MV est l’obstacle majeur à l’implication des soignants à ce système. L’objectif de cette étude est de déterminer les connaissances, attitudes et pratiques des médecins du CHU quant à la mise en place du système de MV.

**Méthodes:**

Etude descriptive de type CAP (connaissances, attitudes et pratiques) transversale auprès de tous les médecins titulaires exerçant au CHU de Sousse (Tunisie) qui sont utilisateurs des dispositifs médicaux (DMs) lors de leur pratique d’activité de soin. Un questionnaire auto-administré, préétabli et pré-testé a été établi. Saisie et l’analysée des données par logiciel SPSS20.0.

**Résultats:**

Le taux de réponse de 51,9 % (183/95), un manque des connaissances relatif à la MV a été rapporté. Plus de la moitié des répondants ne connaissent pas le correspondant local de son établissement et l’existence d’un formulaire standardisé de signalement. Concernant les attitudes, 89,5 % notifient l’intérêt de mise en place du système de MV et 37,5 % reconnaissent que le signalement doit émaner du soignant constatant l’incident. Pour les pratiques, la majorité confirment l’absence d’une gestion organisée de la maintenance des DMs dans leurs services. 90,5 % expriment leurs souhaits de recevoir une information mais peu d’entre eux expriment leurs désirs de suivre une formation (57,9 %).

**Conclusion:**

Un manque d’information et de formation dans un domaine pourtant sensible et devant être lourdement réglementé est soulevé. La promulgation de textes réglementaires est nécessaire afin de promouvoir le secteur des DMs et garantir la sécurité sanitaire du patient et de l’utilisateur.

## Abstract (en)

**Introduction:**

The University Hospital Farhat Hached Sousse (Tunisia), has implemented a device-vigilance (DV) system, according to ANCSEP (National Agency of the Sanitary and Environmental Control of Products) guidelines, in order to manage the risk more effectively in hospital and to improve the quality and safety of patient care. In Tunisia the lack of regulation regarding device vigilance is the major obstacle to caregiver vigilance. The objective of this study is to establish the knowledge, attitudes, and clinical practice of University Hospital physicians regarding the implementation of the DV system.

**Methods:**

We conducted a descriptive cross-sectional study of KAP (knowledge, attitudes and practices) among all the physicians working at the University Hospital Farhat Hached Sousse (Tunisia) who were users of medical devices (MDs) in the practice of their profession. A self-administered, pre-established and pre-testing questionnaire was developed. Data were collected and analyzed using SPSS20.0 software.

**Results:**

The response rate was 51.9 % (183/95). A lack of knowledge about DV has been reported. More than half of the respondents didn’t know the local correspondent of health establishment and the existence of a standardized vigilance reporting form. Regarding the attitudes, 89,5% express their interest in setting up a DV system and 37,5% acknowledged that the vigilance reporting form should be filled by the caregiver notifying the incident. Regarding the procedures, the majority of physicians confirmed the absence of an organized maintenance management of the MDs in the practice of their services. 90.5% express their wishes to receive information but few of them express their wishes to receive proper training (57.9%).

**Conclusion:**

A lack of information and training in a sensitive field which need to be heavily regulated has been a topic of discussion. The promulgation of regulatory texts is necessary in order to promote MD sector and guarantee the safety of patient and their users.

## Introduction

Malgré l’existence de systèmes de contrôle des dispositifs médicaux (DMs) avant leur mise sur le marché de qualités et performants, l’utilisation des (DMs) n’a jamais pu être exempte de risque. De plus, si certains risques sont prévus, identifiés, évalués et acceptés avant la mise sur le marché, certains événements indésirables, imprévisibles, inconnus et parfois graves, peuvent apparaître après une large utilisation des DMs [1]. Par ailleurs, la gestion des risques en milieu de soin, représente une préoccupation importante pour les systèmes de santé d’autant plus croissante que les soins s’intensifient, se diversifient et se spécialisent. Elle associe aussi bien la veille que la vigilance sanitaire en identifiant les risques et la gravité des incidents d’utilisation des DMs par leur recensement pour pouvoir les prévenir ultérieurement [2]. En Tunisie un projet de loi organisant et structurant le système de matériovigilance (MV) est en cours de concrétisation. Néanmoins, ce système fonctionnel est restreint à quelques établissements de santé engagés à l’application des recommandations de l’ANCSEP (Agence Nationale de Contrôle Sanitaire et Environnemental des Produits) malgré l’absence de réglementation spécifique. Le CHU Farhat Hached de Sousse (Tunisie) fait partie de ces quelques établissements qui intègrent la gestion des risques associés aux soins, par la mise en place d’un système de MV depuis 2013 selon les recommandations de l’ANSCEP. C’est dans ce cadre que nous avons mené ce travail à fin d’établir un état des lieux sur les connaissances, attitudes et pratiques des médecins du CHU Farhat Hached concernant la mise en place du système de MV. Notre objectif secondaire était de déterminer les attentes et les propositions du personnel médical relatif à ce dispositif. Dans le but de les impliquer dès les premières étapes de la mise en place de ce système pour en garantir le succès.

## Méthodes

Le CHU ‘’FARHAT HACHED’’ de Sousse (Tunisie) est composé de 26 services médicaux, 4 services chirurgicaux, 9 laboratoires et doté d’une capacité hospitalière de 698 lits. Les professionnels de la santé titulaires exerçant à l’hôpital sont au nombre de 1414 dont 241 médecins. Nous avons menés observationnelle descriptive transversale et exhaustive menée durant le mois de Janvier 2015 auprès de tous les médecins exerçant dans 24 services dispensant un soin (diagnostic ou thérapeutique) aux patients au CHU Farhat Hached de Sousse (Tunisie). Tous les médecins titulaires (N=183) tous grades et spécialistes confondus exerçant dans les services ayant des activités de soins ont été inclus. L’étude a été menée à l’aide d’un questionnaire pré établi, pré testé et auto administré. Le questionnaire comporte 30 questions réparties en 5 parties. Une lettre d’information à été adressée aux chefs des services présentant l’étude, expliquant son cadre et exposant ses objectifs avant le lancement du questionnaire. Les médecins faisant partie de notre population à l’étude ont été contactés et informés des objectifs du travail en leur assurant l’anonymat des résultats.

La saisie et l’analyse des résultats ont été réalisées à l’aide du logiciel SPSS 18. Les variables quantitatives ont été présentées sous forme de moyennes plus ou moins leurs écart-types. Les variables qualitatives ont été présentées sous forme de pourcentages. Pour chaque estimation, un intervalle de confiance a été calculé selon la formule classique. Lorsque les conditions d’application de la formule classique n’étaient pas remplies (np < 5 ou n(1-p) <5), la procédure de Wilson avec correction de continuité a été utilisée [3].

## Résultats

Le taux de réponse globale est de 51,9 % (Il est 48,02 % pour les médecins titulaires universitaires (n1 = 152), de 72,41 % pour les médecins titulaires de la santé publique (n2 = 29) et de 50% pour les médecins des hôpitaux (n3 = 2)). L’âge moyen des participants était de 42,55 (±8,07) ans avec des extrêmes de 30 a 60 ans. Nous avons noté une prédominance féminine avec un sex ratio de 0,46. Les caractéristiques générales des médecins enquêtés sont rapportées dans le Tableau 1. Les connaissances des répondants à l’étude concernant le dispositif de MV, leurs attitudes et leurs pratiques ainsi que attentes suite à la mise en place de ce système sont détaillés dans le Tableau 2.

**Tableau 1:** Caractéristiques généraux des répondants (n = 95)

Variables		Effectifs	Pourcentages
Sexe	masculin	30	31,6%
	féminin	95	68.4%
Statut d’exercice	universitaire	73	76,8%
	santé publique	21	22,1%
	hôpitaux	1	1,1%
Ancienneté professionnelle	moins de 10 ans	46	48,8%
	10 à 20 ans	36	37,9%
	plus de 20 ans	13	13,7%
Service d’exercice	Médical	77	81,1%
	Chirurgical	18	18,9%

**Tableau 2:** Répartition des participants selon leurs connaissances, attitudes, pratiques et attentes relatives au système de MV (n=95)

Concepts			Effectifs (%)	IC à 95 %
Connaissances	Générales concernant le système MV	Avoir entendu parler de MV	35 (68,4%)	(58,9 à 77,9 %)
		Avoir été informé du dispositif national de MV	28 (29,5%)	(21,1 à 38,9 %)
	Instance responsable et réglementation	La présence de réglementation nationale sur la MV	32 (33,7)	(25,3 à 43,2 %)
		Instance nationale responsable	62 (65,3%)	(54,7 à 74,7 %)
		L’obligation du signalement	43 (45,3%)	(35,8 à 55,8 %)
		L’existence de formulaire standardisé de signalement	45 (47,4%)	(36,8 à 57,9 %)
	Aspects organisationnels	Correspondant local de MV	66 (69,5 %)	(60 à 77,9 %)
		Formation organisés dans le cadre du dispositif de MV	18 (18,9%)	(11,6 à 26,3 %)
Attitudes	Intérêt et initiation au dispositif de MV	Il est intéressant de mettre en place ce système de MV	85 (89,5%)	(83,2 à 94,7 %)
		Les soignants disposent des notions de bases relatives à la MV	9 (9,5%)	(4,2 à 15,8 %)
	Estimations des incidents liées aux DMs	graves	39 (41,1%)	(30,6 à 50,5 %)
		fréquents	27 (28,4%)	(18,9 à 37,9 %)
	Perception du personnel responsable du signalement	le soignant sans tenir compte du grade	37 (38,9%)	(29,5 à 49,5 %)
		Le chef de service	33 (34,7%)	(26,3 à 44,2 %)
		le surveillant du service	19 (20%)	(12,6 à 28,4 %)
Pratiques	Procédures spécifiques au service	Présence de gestion organisée des DMs dans le service d’exercice	26 (27,4%)	(18,9 à 36,8 %)
		Organisation de la formulation de déclaration au titre de MV	8 (8,4%)	(3,2 à 13,7 %)
	Formation et information	Participation à une formation sur la MV	8 (8,4%)	(3,2 à 15,8%)
		Réception des informations des alertes sanitaires	41 (43,2%)	(33,7 à 52,6%)
		Réception d’un retour d’information	34 (35,8%)	(27,4 à 95,8%)
Attentes	Appui pour un meilleur usage du dispositif	Recevoir plus d'information	86 (90,5%)	(84,2 à 95,8%)
		Participer à une formation spécifique	55 (57,9%)	(47,4 à 67,4%)
		Disposer d’un accompagnement lors des premiers signalements	86 (90,5%)	(84,2 à 95,8%)
	Aspects organisationnels locales	Mise en place et fonctionnalisé une unité de MV dans le CHU	92 (96,8%)	(92,6 à 100 %)
		Identification dans chaque service, d’un référent de MV	90 (94,7%)	(89,5 à 98,9 %)

## Discussion

### Discussion de la méthodologie

Notre étude a taux de participation de 51,9%. Ce taux peut être considéré comme satisfaisant au regard de celui généralement constaté dans ces types d’enquêtes. En effet, une enquête française relative aux connaissances du cadre médicale a propos du dispositif de MV retrouve un taux de réponse de 37% [4]. Notre travail a eu le mérite de constituer un moyen de sensibilisation et d’initiation du personnel soignant médical au système de MV dès les premières étapes de sa mise en place.

Un biais de déclaration peut être posé par notre étude puisque l’évaluation des connaissances, attitudes et pratiques s’est faite à l’aide d’un questionnaire auto-administré, auto-rempli. Par ailleurs, et afin de pallier à ce biais, nous avons prévu, d’une part une lettre d’information qui a précédé l’étude et qui a été adressée aux chefs de services et d’autre part, un préambule dans le questionnaire expliquant clairement l’objectif de l’étude. De plus, la charge du travail et le manque de disponibilité des médecins (services à vocation chirurgicale, réanimation et les unités de soins intensives) constituent les contraintes de terrain non négligeable au déroulement de notre enquête.

### Discussion des résultats

#### Profils des enquêtés

Notre étude révèle, à travers les caractéristiques de ses répondants (âge moyen), une meilleure implication des jeunes médecins titulaires de l’établissement à ce nouveau concept de gestion des risques en milieu de soin. Notre étude montre un manque de connaissance auprès des enquêtés du mode de fonctionnement et de l’organisation du dispositif avec son impact sur les pratiques. Dans ce cadre, Danchin rapporte l’avis de plusieurs auteurs qui pensaient que beaucoup de médecins, partout dans le monde, étaient encore dans l’ignorance ou la mauvaise connaissance de la démarche, même dans les pays ayant un système organisé, réglementé et structuré par la loi [5].

*Connaissances des enquêtés* Bien qu’aucune étude tunisienne n’ait auparavant évalué les connaissances des médecins relativement à la MV, l’ANCSEP, par plusieurs moyens d’information mais surtout à travers le réseau nationale en santé de messagerie par Internet « rns » et ses correspondants locaux, précise que la MV a pour objet la surveillance des incidents ou des risques d’incidents résultant de l’utilisation des DMs après leur mise sur le marché [1].

En France, la MV est définie à l’article 8 de la directive 93/42/CEE relative aux DMs [6]. De plus, c’est l’article R. 665-48 du code de la santé publique français qui fixe l’organisation de la MV [7]. Aux Etats Unis c’est le « Code of Federal Regulation » regroupant les aspects qualité et réglementation dans son chapitre 803 qui aborde spécifiquement la MV [8]. Une coordination mondiale entre les différents pays (Union Européenne, États-Unis, Canada, Australie et Japon) a été développée depuis 1992 grâce au groupe de travail « Global Harmonization Task Force » qui œuvre, en collaboration avec l’Organisation Mondiale de la Santé, à réunir les représentants des autorités compétentes adhérentes ainsi que des représentants de l’industrie afin de décider des nouvelles orientations et de l’harmonisation de la réglementation dans le but d’améliorer la sécurité des patients et accroître l´accès à des soins efficaces et à des technologies médicales bénéfiques à travers le monde [4, 9]. Notre étude révèle un manque de connaissance relatif au correspondant local de l’établissement et sa mission précise. Dans les pays ou ce dispositif est réglementé, cette mission est dictée par les textes juridiques normatifs spécifiant exactement son rôle [7].

Si on se réfère au modèle français, Jayet dans son étude précise que la profession du correspondant local de MV dépend étroitement du type d’établissement de santé [2]. La reconnaissance du caractère obligatoire des signalements et l’existence d’un formulaire unique et standardisé de signalement des incidents ou risque d’incident en MV n’ont été reconnu que seulement par moins de la moitié des participants à l’étude. Dans ce sens, Canivet rapporte qu’habituellement c’est le correspondant local de MV qui est chargé généralement de transmettre à l’autorité centrale toute déclaration d’incident ou risque d’incident faite auprès de lui et de conduire les enquêtes relatifs à la sécurité d’utilisation des DMs. Par ailleurs, tout soignant, faisant la constatation ou ayant connaissance d’incident ou risque d’incident mettant en cause un DMs doit le déclarer auprès du correspondant local de MV [10].

Néanmoins, dans certains pays, comme les Etats Unis, ce sont les fabricants et les importateurs de DMs qui ont l’obligation de signaler tout incident ou risque d’incident [11]. La déclaration des incidents par les professionnels de santé est obligatoire dans certains pays (France, États-Unis), tandis que d’autres pays (Angleterre et Australie) comptent sur leur participation volontaire [4, 8, 9].

Notre travail révèle un manque d’information relative à l’existence du système de MV et de la formation spécifique dédiée à la MV au niveau de l’établissement. Ailleurs, dans les pays disposant de dispositifs réglementés, on peut l’attribuer aux sources d’information qui sont, en outre, souvent peu attrayantes car trop techniques pour le lecteur néophyte : Journal officiel, guide de la MV, guidelines on a medical devices vigilance system [1, 7, 12, 13].

#### Attitudes des enquêtés

Le manque de perception de l’intérêt de la mise en place de ce système dans notre établissement peut être expliqué par la faible incarnation auprès de certains soignants d’une culture de qualité et sécurité des soins et qui doit être renforcée lors de l’usage des DMs [13]. Les enquêtés estiment comme exceptionnel les incidents ou risque d’incidents relevant de la MV susceptibles de survenir dans leurs services d’exercice, cependant, ils les qualifient de grave dans presque la moitié des cas de survenue.

Le code de santé publique français précise les incidents qualifiés comme graves [7]. En Tunisie, Frikha rapporte dans son étude que 70% des incidents notifiés ont été jugés comme graves, alors que le pronostic vital n’a jamais été mis en jeu [13]. Dans le même sens, l’évaluation proprement dite de l’incident au niveau du service, émetteur du signalement, fait encore défaut [13]. En France, Ancellin aborde les difficultés rencontré pour l’évaluation des incidents [14]. Par conséquent, le correspondant local peut faire appel, quand il le juge nécessaire, aux spécialistes aidant l’évaluation de l’incident [7]. Il faut bien distinguer lors des évaluations les vrais incidents des mésusages qui sont des sources d’erreurs [7]. D’ailleurs, les premiers bilans de MV en France ont montré des résultats évoluant dans le même sens que les études rapportés par Ancellin et Cazalaà qui soulignent que la majorité de leurs incidents correspondent plutôt à des erreurs d’utilisation [14, 15]. Beydon rapporte que de nombreuses déclarations sont exclues du champ de la MV par l’Afssaps (Agence Française de Sécurité Sanitaire des Produits de Santé) parce que d’autres facteurs sont mis en évidence : mauvaise utilisation, contexte clinique, particularités liées aux patients, il précise dans le même sens, qu’un taux de déclarations inutiles par période pouvant atteindre 30 % [16]. Regeasse et Gross insistent sur la confusion des procédures de MV et de maintenance [4, 11]. Les enquêtés précisent que le personnel le plus apte devant procéder au signalement est tout soignant sans tenir compte de son grade, constatant l´incident ou risque d´incident ainsi la législation française tout comme l’ANCSEP précisent les missions reconnues aux correspondants locaux [1, 7].

Bien que l’ANCSEP dès la mise en place du dispositif n’ait pas limité les déclarations aux incidents graves, les premiers bilans enregistrés et publiés sont très faibles [1, 13]. Dans le même sens l’agence Nationale de Sécurité du Médicament et des Produits de Santé Française (ANSMPSF) rapporte 1222 signalements en 1996 et 4182 en 1998 [2]. Cette constatation s’explique par le fait, qu’en France, la réglementation stipule que tout incident ou risque d’incident grave doit être signalé et une condamnation pénale a même été prévue [7].

Dans le même cadre, Beydon note la hausse des signalements d’incidents chaque année en France ce qui traduit un effort d’implication face à la nouvelle réglementation. En 2002, le nombre de déclarations a légèrement diminué car Le système n’a pas encore atteint sa maturité [16]. Ancelin confirme ce fait en comparant la situation française et américaine sur un exemple : entre 1985 et 1999, sur les 2,5 millions de lits médicaux aux États-Unis, 371 incidents (dont 228 décès) mettant en cause des barrières de lit ont été rapportés [17]. De tels événements surviennent aussi en France. Ils ont pourtant été longtemps peu, voir pas du tout signalés [17]. Danchin ajoute que certains professionnels de santé ont, parfois, quelques réticences à effectuer des déclarations par crainte de « tracasseries » ultérieures [5]. Ils redoutent que l’expertise établisse que les effets nocifs constatés sur le patient résultent d’une mauvaise utilisation du DM et non de sa défaillance.

*Pratiques des enquêtés* Un manque de participation à des formations relatives à ce dispositif de MV a été noté dans notre étude. Des études françaises démontrent qu’un signalement ne peut être opérationnel que si les utilisateurs aient été préalablement formés et sensibilisés à la MV et que le circuit de déclaration des alertes soit connu de tous [4, 7, 14]. Les participants à notre étude rapportent qu’ils n’ont pas procédé à une déclaration au titre de la MV dans la quasi majorité des cas (91,6 %). Par ailleurs, un bilan national tunisien des activités a montré des disparités régionales en termes de signalement émanant des établissements de santé. Les blocs opératoires et les services d’anesthésie et réanimation ont été les services les plus déclarants. En effet, il s’agit de services hospitalisant des patients dont le pronostic vital est mis en jeu et chaque incident peut s’avérer fatal [13]. Cela est confirmé par l’étude de Cazalaa relative au bilan de la MV dans l’hôpital Necker [15] où le nombre d’incidents émis par le service de réanimation dépasse de loin celui émis par les autres services. De même, l’étude réalisée en 2009, par Thevenin, à l’hôpital Foch a démontré que 60% des événements déclarés proviennent du bloc opératoire central [18]. Regeasse dans une étude française rapporte que le manque d’implication des utilisateurs au signalement freine le fonctionnement du système qui repose essentiellement sur les déclarations [4].

Notre étude montre, dans plus de la moitié des cas, un manque, d’une part, de la réception des informations concernant les alertes sanitaires et, d’autre part, de retour de l’information. Frikha dans son étude rapporte que l’étape relative au retour de l’information est primordiale dans le système de MV d’autant plus que les CHU ayant reçu une réponse à leurs signalements ont pris désormais l’habitude de signaler, les CHU n’ayant pas effectué d’autres signalements sont ceux dont les délais de traitement de leurs dossiers étaient trop longs [13]. En France, à la suite d’un signalement, le correspondant local reçoit un accusé de réception de l’Afssaps dans un délai moyen de deux jours [15]. Il doit tenir à jour un fichier des incidents signalés et des suites qui lui sont données et en informer les intervenants concernés [14]. Beydon estime cependant que le retour d’information est, dans la pratique, peu organisé [16]. Regeasse dans son étude constate que le retour d’information est plus important vers les personnels soignants dans les grands services (trois médecins ou plus) que dans les petits. Ceci peut s’expliqué par le fait que dans les grandes unités de soins, une personne peut se charger particulièrement des tâches administratives alors que le praticien isolé, appelé à tout faire, peut considérer comme non prioritaire les courriers relatifs au suivi d’une déclaration [4]. Amoore précise, dans la même logique, que les bulletins d’alerte sont distribués par l’échelon central via le correspondant local de l’établissement. La moitié seulement des médecins enquêtés déclarent en avoir reçu. Lorsqu’il s’agit de recommandations d’utilisation, il est pourtant primordial que les professionnels de santé soient bien informés afin que la leçon des incidents précédents soit tirée [19].

#### Les attentes et propositions

Presque tous les participants à l’étude ont formulé, leur souhait de recevoir plus d´informations, de disposer d’une unité de MV dans l’établissement de santé et de posséder, dans chaque service, d’un référent de MV. La meilleure organisation de l’information et la planification de la formation ont été rapportées dans plusieurs études dont celles menés en France par Amoore [19] et en Australie par Beech [20]. Cependant, le souhait de suivre une formation spécifique a été peu mentionné ce qui peut être expliqué, d’une part, par les contraintes liées à la charge de travail et l’indisponibilité des enquêtés, et d’autre part, par les méthodes, parfois, peu attrayantes et classiques des formations

#### Les recommandations

En Tunisie, l’ANCSEP a pu fédérer les efforts afin de lancer le système de MV dans les grands établissements de santé répartis sur le territoire national, malgré que le démarrage semble être difficile. Le cas du CHU Farhat Hached ne diffère pas de la tendance de la situation nationale. Plusieurs actions restent à développer et certains engagements demeurent à prendre. Il s’agit notamment de: accélérer impérativement la promulgation des textes juridiques; oeuvrer à regrouper davantage les vigilances sanitaires à tous les échelons (mise en commun des moyens utilisés et harmonisation des procédures); affirmer le caractère obligatoire de la MV en l’intégrant dans le cursus de formation médicale paramédicale; programmer un plan de communication permettant de couvrir tous les professionnels de santé; oeuvrer à l’élaboration d’un guide national de la MV qui constituera un bon support de base pour l’enseignement, l’information et la sensibilisation; formaliser l’organisation et la structuration de la MV; oeuvrer à une meilleure implication de l’administration argumentée par l’avènement de l’accréditation et la certification des établissements de santé qui comporte dans leurs critères les « vigilances sanitaires »; créer au niveau local un comité de MV pluridisciplinaire au sein de l’établissement de santé; renforcer la formation la sensibilisation et l’information des compétences des correspondants locaux; informatiser l’organisation du système; créer un troisième échelon dans le système en constituant ainsi un réseau de relais de référent de MV dans chaque unité de soin de l’établissement de santé; prévoir une organisation de la gestion des incidents et des alertes relevant de la MV lors de l’absence du correspondant de l’établissement (les week-ends, les jours fériés et les congés) du fait que la déclaration des incidents sans délai s’impose à tout professionnel des établissements de santé.

## Conclusion

L’usage des DMs dans les soins n’est jamais exempte de risques et peut mettre en jeu aussi bien la sécurité des patients que celle des soignants. La meilleure gestion des risques en milieu hospitalier et l’amélioration de la qualité et de la sécurité des soins exige la meilleure organisation des vigilances sanitaires ce qui impose, la mise en place d’un dispositif de MV. Dans ce cadre, l’ANCSEP a initié un système de MV pour la Tunisie. Le démarrage de cette activité a été à la fois timide mais aussi difficile. La situation de notre CHU ne diffère pas de la situation nationale ce qui nous a incité a mené ce travail apportant un état des lieux relatif à ce dispositif afin de mieux cibler nos actions dans une perspective d’optimiser les chances de réussite de ce système. Ainsi nous proposons des actions, qui sont relativement simples, à mettre en œuvre sur le plan local. Cependant, l’inférence de leur mise en application, à l’échelle nationale, dans tous les hôpitaux tunisiens reste toujours souhaitable. Une enquête analogue à la nôtre pourrait ensuite être engagée afin de permettre d’évaluer l’efficacité des mesures éventuellement entamées. La réussite de la fonctionnalité du système de MV doit être soutenue par des textes réglementaires, ce qui n’est pas encore le cas en Tunisie. La promulgation d’un texte de loi pour constituer l’assise du système de MV est impérative. Entre temps, l’ANCSEP et les correspondants locaux désignés dans les établissements de santé doivent assumer pleinement leurs rôles dans ce dispositif aussi bien à l’échelle locale que régionale.

### Etat des connaissances actuelles sur le sujet

- Système qui renforce la gestion des risques liées aux soins et qui améliore la qualité et la sécurité des soins;
- Système qui renforce les vigilances sanitaires en améliorant la gestion des incidents et l’anticipation sur les risques d’incidents liées à l’usage des dispositifs médicaux avec possibilités de mettre en commun les moyens utilisés et harmonisés de certaines procédures;
- Système qui prépare à l’accréditation et à la certification des établissements de santé d’autant plus qu’en Tunisie le ministère de la santé vient de mettre en place récemment l’INAS (Instance Nationale d’accréditation en santé).

### Contribution de notre étude à la connaissance

- Promulguer nécessairement un texte de loi réglementant et organisant la matério-vigilance et permettant, ainsi, une responsabilisation et une obligation pour s’y mettre à l’application du système;
- Affirmer le caractère médical de la matério-vigilance en l’intégrant dans le cursus de formation académique de base et continue des médecins afin d’améliorer la perception du risque lié à l’usage des dispositifs médicaux et de combler les manques de formation soulevés et leurs impacts sur les attitudes et pratiques des médecins concernés;
- Etablir un plan de communication pour renforcer l’information et la sensibilisation quant à l’intérêt de mettre en place le système et les modalités de son usage.
